# Supplementary material for: Chronic Kidney Disease Is Associated with High Mortality Risk in Patients with Diabetes after Primary Shoulder Arthroplasty: A Nationwide Population-Based Cohort Study
Source: Diagnostics (Basel). 2021 May 1;11(5):822. doi: 10.3390/diagnostics11050822 (PMC8147363; doi:10.3390/diagnostics11050822)
Supplement: Supplementary file 1 [file diagnostics-11-00822-s001.zip › diagnostics-1193982-supplementary.pdf]

Table S1 ICD-9-CM codes used for diagnosis in the current study

Variable	ICD-9-CM code
Type 2 diabetes mellitus	250.xx excluding type 1 DM
Type 1 diabetes mellitus	250.01, 250.03, 250.11, 250.13, 250.21, 250.23, 250.31, 250.33, 250.41, 250.43, 250.51, 250.53, 250.61, 250.63, 250.71, 250.73, 250.81, 250.83, 250.91, 250.93
Cardiac dysrhythmia	427.xx
Chronic kidney disease	580.xx–589.xx, 403.xx–404.xx, 016.0x, 095.4x, 236.9x, 250.4x, 274.1x, 442.1x, 447.3x, 440.1x, 572.4x, 642.1x, 646.2x, 753.1x, 283.11, 403.01, 404.02, 446.21
Chronic obstructive pulmonary disease	491.xx, 492.xx, 496.xx
Coronary heart disease	410.xx–414.xx
Dementia	290.xx, 294.xx
Dialysis	585.xx (Catastrophic illness card)
Heart failure	428.xx
Hyperlipidemia	272.xx
Hypertension	401.xx–405.xx
Immune diseases	710.0, 710.1, 714.0, 710.4, 710.3, 446.0, 446.2, 446.4, 446.5, 443.1, 446.7, 136.1, 694.4, 710.2, 555.xx, 556.xx, 714.30–714.33
Implant infection	996.66, 996.67
Malignancy	140.xx–208.xx (Catastrophic illness card)
Multiple trauma	959.99
Old myocardial infarction	410.xx, 412.xx
Osteoporosis	733.xx
Stroke	430.xx–432.xx, 433.xx–437.xx,

ICD-9-CM, International Classification of Diseases, Ninth Revision, Clinical Modification.

Table S2 Distribution of renal function status for diabetic patients who received shoulder arthroplasty across 1998-2013

Year	Non-CKD	Non-dialysis CKD	Dialysis
1998	132 (97.1)	4 (2.9)	0 (0.0)
1999	133 (84.7)	19 (12.1)	5 (3.2)
2000	138 (78.4)	33 (18.8)	5 (2.8)
2001	140 (71.8)	51 (26.2)	4 (2.1)
2002	157 (75.8)	45 (21.7)	5 (2.4)
2003	174 (71.6)	57 (23.5)	12 (4.9)
2004	239 (73.1)	82 (25.1)	6 (1.8)
2005	212 (67.7)	87 (27.8)	14 (4.5)
2006	223 (68.6)	87 (26.8)	15 (4.6)
2007	214 (67.7)	86 (27.2)	16 (5.1)
2008	231 (64.0)	114 (31.6)	16 (4.4)
2009	252 (64.1)	111 (28.2)	30 (7.6)
2010	227 (65.6)	100 (28.9)	19 (5.5)
2011	196 (56.3)	115 (33.0)	37 (10.6)
2012	198 (63.9)	93 (30.0)	19 (6.1)
2013	176 (60.7)	90 (31.0)	24 (8.3)
Total	3042 (68.5)	1174 (26.4)	227 (5.1)

Abbreviations: CKD, chronic kidney disease;
Data were given as frequency (percentage);
p trend <0.001.

Table S3 Subgroup analysis of late outcomes by study period

	Number of events (%)			Adjusted HR or SHR (95% CI) ‡		
Outcome / Period	Non-CKD	Non-dialysis CKD	Dialysis	Non-dialysis CKD *vs.* Non-CKD	Dialysis *vs.* Non-CKD	Dialysis *vs.* Non-dialysis CKD
Infection event						
Year 1998-2005	147 (11.1)	33 (8.9)	9 (17.6)	0.90 (0.61–1.34)	1.65 (0.83–3.28)	1.83 (0.87–3.88)
Year 2006-2013	81 (4.7)	42 (5.4)	17 (9.8)	1.18 (0.81–1.73)	1.85 (1.06–3.22)	1.56 (0.87–2.81)
All-cause readmission						
Year 1998-2005	1,221 (92.5)	352 (94.6)	48 (94.1)	1.61 (1.41–1.85)	2.53 (1.93–3.32)	1.57 (1.18–2.10)
Year 2006-2013	1,161 (67.8)	581 (74.0)	138 (79.8)	1.18 (1.08–1.29)	1.48 (1.26–1.73)	1.25 (1.06–1.48)
All-cause mortality						
Year 1998-2005	763 (57.6)	287 (75.9)	50 (98.0)	1.77 (1.54–2.04)	4.36 (3.25–5.85)	2.46 (1.81–3.35)
Year 2006-2013	428 (24.9)	268 (33.7)	93 (52.8)	1.44 (1.24–1.69)	3.80 (3.01–4.80)	2.63 (2.07–3.36)

Abbreviations: CKD, chronic kidney disease; HR, hazard ratio; SHR, subdistribution hazard ratio; CI, confidence interval;
Data were given as frequency (percentage);
‡ The model was adjusted for all covariates listed in Table 1 in which the follow-up year was replaced with the index date.
